# A Lifespan Perspective on Entrepreneurship: Perceived Opportunities and Skills Explain the Negative Association between Age and Entrepreneurial Activity

**DOI:** 10.3389/fpsyg.2017.02015

**Published:** 2017-12-01

**Authors:** Clarissa Bohlmann, Andreas Rauch, Hannes Zacher

**Affiliations:** ^1^Work and Organizational Psychology, Institute of Psychology, Leipzig University, Leipzig, Germany; ^2^The University of Sydney Business School, University of Sydney, Sydney, NSW, Australia; ^3^School of Management, Queensland University of Technology, Brisbane, QLD, Australia

**Keywords:** age, entrepreneurship, lifespan, perceived skills, perceived opportunities

## Abstract

Researchers and practitioners are increasingly interested in entrepreneurship as a means to fight youth unemployment and to improve financial stability at higher ages. However, only few studies so far have examined the association between age and entrepreneurial activity. Based on theories from the lifespan psychology literature and entrepreneurship, we develop and test a model in which perceived opportunities and skills explain the relationship between age and entrepreneurial activity. We analyzed data from the 2013 Global Entrepreneurship Monitor (GEM), while controlling for gender and potential variation between countries. Results showed that age related negatively to entrepreneurial activity, and that perceived opportunities and skills for entrepreneurship mediated this relationship. Overall, these findings suggest that entrepreneurship research should treat age as a substantial variable.

## Introduction

*Entrepreneurship* is defined as the discovery, evaluation, and exploitation of opportunities to create new and useful products and services (Shane and Venkataraman, 2000). In times of economic uncertainty and an aging workforce (cf. Funken and Gielnik, 2016), entrepreneurship may become a promising career path, particularly for older adults (Rogoff, 2009; Kulik et al., 2014; Halvorson and Morrow-Howell, 2016). However, the attractiveness of entrepreneurship compared to salaried employment might vary with age, because abilities and motivations related to entrepreneurial activity are likely to change over the lifespan (e.g., Ainsworth, 2015). This study therefore aims to investigate the relationship between age and entrepreneurial activity, while examining perceived entrepreneurial opportunities and skills as mediators of this association.

The importance of age for entrepreneurial activity is grounded in the lifespan perspective (Baltes, 1987), according to which development is a lifelong process characterized by both gains and losses in psychological characteristics. As people get older, some abilities such as physical strength tend to decline on average, whereas other abilities such as crystallized intelligence (e.g., knowledge, skills) are maintained or increase. Research in the field of lifespan developmental psychology (Baltes and Baltes, 1990; Carstensen et al., 1999; Lang and Carstensen, 2002) further suggests age-related changes in motives and goal orientations (Kanfer et al., 2013; Truxillo et al., 2015; Hertel and Zacher, 2018). These changes could influence entrepreneurial activity as a form of goal-oriented action.

Researchers have suggested that the relationship between age and entrepreneurial activity is generally negative (Lévesque and Minniti, 2006). However, the processes underlying this relationship are largely unknown, and the links among age, age-related characteristics, and entrepreneurial activity are therefore not well understood. Interestingly, many studies have assumed that age plays a role for entrepreneurship by including it as a control variable in the prediction of entrepreneurial activity, but have refrained from treating it as a substantial variable. Exceptions are the conceptual paper by Lévesque and Minniti (2006), two studies by Gielnik et al. (2012, 2017), in which individuals’ focus on opportunities mediated the negative relationship between age and venture growth, and a study by Minola et al. (2016), in which the results revealed an inversely U-shaped association between age and both perceived feasibility and desirability belief regarding self-employment.

**FIGURE 1:**
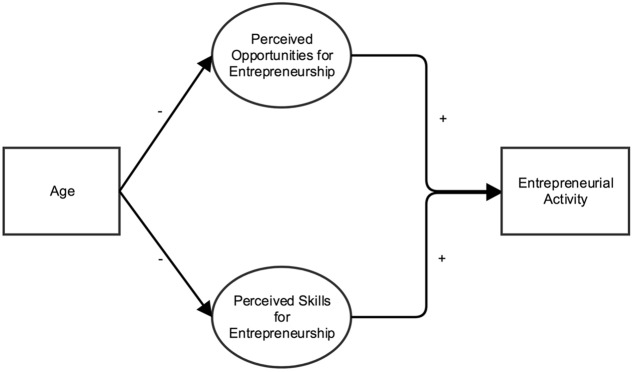
Conceptual model of how age relates to entrepreneurial activity through perceived opportunities and perceived skills for entrepreneurship.

With the present study, we contribute to this literature by adopting a lifespan perspective to investigate the role of age for entrepreneurship. Using data from the 2013 Global Entrepreneurship Monitor (GEM), a large scale international survey study, we answer calls for research on the factors that explain how and why age relates to entrepreneurial activity (Lévesque and Minniti, 2006; Ainsworth, 2015). Specifically, we investigate perceived opportunities for entrepreneurship (i.e., perceptions of the availability of situations in which new goods, services, raw materials, markets and organizing methods can be introduced through the formation of new means, ends, or means-ends relationships; Shane and Venkataraman, 2000), and perceived skills for entrepreneurship (i.e., perceptions of the amount of skills people have in order to act entrepreneurially) as mediators of the relationship between age and entrepreneurial activity (see **Figure 1**). Thus, we build on research highlighting the importance of individual perceptions for entrepreneurship (Arenius and Minniti, 2005). We find that both perceived opportunities and skills partially explain the negative and weakly curvilinear relationship between age and entrepreneurial activity. Ultimately, examining these mediators of the relationship between age and entrepreneurial activity helps disentangle the complex role of age for entrepreneurship and to uncover the mechanisms through which age relates to entrepreneurial activity (Bohlmann et al., 2017). Based on these findings, entrepreneurship training could be tailored to the characteristics of different age groups.

## Entrepreneurial Activity and Age

Different entrepreneurship patterns have been observed in relation to age (Ainsworth, 2015). In general, it can be said that acting entrepreneurially requires the recognition and exploitation of business opportunities. This process takes time and is affected by individual characteristics such as physical strength to overcome obstacles, as well as cognitive skills to solve problems associated with the business and to complete day-to-day activities.

This role of age for entrepreneurial activity can be explained using theorizing by Lévesque and Minniti (2006), as well as the lifespan perspective (Baltes, 1987). Lévesque and Minniti (2006) argue that a negative relationship between age and entrepreneurial activity is due to opportunity costs of time. That is, as people age, they perceive that they have less time remaining in their life (Carstensen et al., 1999). This perception may, in turn, influence entrepreneurial activity, which generally requires the recognition and exploitation of business opportunities, and thus time, to generate returns. More specifically, as people age, they perceive that they have less time to rely on the uncertain returns from entrepreneurship, and are therefore less willing to translate their business ideas into action. The result may be a decreasing interest in entrepreneurial activity with age as individuals tend to discount activities not yielding imminent returns, and instead prefer instant payoffs as they age (Carstensen et al., 1999). Ultimately, one could say that due to their limited time remaining, older people perceive their opportunities for generating income with entrepreneurship as limited. This observation is supported by research showing that the interest in becoming an entrepreneur decreases with age (e.g., Blanchflower et al., 2001). Moreover, decreasing physical abilities (Spirduso et al., 1995) and fluid intelligence (Salthouse, 2009) with age may lead to lower perceptions of skills for entrepreneurship, and ultimately entrepreneurial activity. Based on this theorizing, we hypothesize that there is a negative relationship between age and entrepreneurial activity.

(1) *Hypothesis 1*: Age relates negatively to entrepreneurial activity.

While goals and motives may change over the lifespan, age itself is merely an umbrella variable that stands for the change associated with the passage of time (Wohlwill, 1970; Zacher, 2015). To better understand the role of time for entrepreneurial activity, it is therefore important to also investigate the mediators of the proposed negative relationship between age and entrepreneurial activity. Two potential mediators, perceived opportunities and skills, will be discussed in the next sections.

### The Role of Perceived Opportunities

Following propositions of theories from the domain of lifespan psychology (Baltes and Baltes, 1990; Carstensen et al., 1999), we argue that entrepreneurial motivation is likely to change when people get older. Importantly, motivational changes are not directly caused by age, but by other age-related factors such as the remaining time and opportunities people perceive in their lives (Carstensen, 2006; Cate and John, 2007; Henry et al., 2017).

The lifespan perspective suggests that this change in people’s perception of opportunities is related to several age-related individual (i.e., internal) and contextual (i.e., external) mechanisms. Regarding internal mechanisms, research has shown an age-related decline of resources such as information processing, perceived time left, and physical stamina (Schulz and Heckhausen, 1996; Baltes and Lang, 1997). As these resources are important to maintain a focus on opportunities, the *perception of opportunities* (i.e., how many new goals, plans, options, and opportunities people believe to have in their personal future; Cate and John, 2007; Zacher and Frese, 2009) is likely to decrease with age. External mechanisms are, for example, age-related norms and environmental constraints that may lower perceived opportunities for entrepreneurship. Norms in Western societies often depict retirement as the better alternative over pursuing new professional opportunities once a certain age is reached (Neugarten et al., 1965; Hershey et al., 2002; Kautonen et al., 2010). Based on these arguments, we hypothesize that the perception of opportunities for entrepreneurship decreases with age.

(1) *Hypothesis 2*: Age relates negatively to perceived opportunities for entrepreneurship.

To act upon a business opportunity, it is first necessary to perceive it (Baron, 2004). Once an opportunity is detected, its actual pursuit often depends on individual goal selection and persistence during goal pursuit (Seijts, 1998; Aspinwall, 2005). According to lifespan psychology, perceiving that many opportunities remain in life is likely to lead to the pursuit of long-term goals. Regarding entrepreneurial activity, those long-term goals may be venture creation or business foundation. In contrast, people perceiving fewer opportunities are likely to pursue short-term goals, such as emotional well-being (Lang and Carstensen, 2002; Carstensen, 2006). Individuals who perceive many remaining opportunities are likely to set more challenging goals, and ultimately apply higher standards to evaluate their goal-accomplishment (Markus and Nurius, 1986; Cross and Markus, 1994). Based on these findings, researchers have concluded that perceiving opportunities fosters the amount of effort and persistence invested in goal pursuit, leading to higher work engagement and performance (Zacher et al., 2010; Schmitt et al., 2013). Consequently, the perception of opportunities for entrepreneurship should positively impact entrepreneurial activity, as people set more ambitious long-term goals and are more likely to pursue these.

An example of the importance of opportunity perception for entrepreneurial activity is entrepreneurship training. In those programs, people are often trained to recognize opportunities. Research by DeTienne and Chandler (2004), for example, showed that after the training, students were better able to identify opportunities, while generating more ideas with higher innovativeness. Increased opportunity recognition, in turn, may heighten entrepreneurial behavior. We therefore assume that perceiving opportunities is positively related to entrepreneurial activity.

(1) *Hypothesis 3*: Perceived opportunities for entrepreneurship relate positively to entrepreneurial activity.

Taking Hypotheses 2 and 3 together, we argue that perceived opportunities mediate the proposed negative relationship between age and entrepreneurial activity. More specifically, due to lower perceptions of entrepreneurial opportunities, older adults are likely to see themselves as less prepared for entrepreneurial activity. They view their time to achieve long-term goals as limited, and thus prefer to maximize present outcomes such as immediate financial returns (Lévesque and Minniti, 2006). In addition, older people have usually achieved their most important personal and business goals, such as desired income (Smallbone and Wyer, 2006), and therefore might not focus on opportunities as much as their younger counterparts. This corresponds to research findings by Curran and Blackburn (2001), which show that the main reasons against entrepreneurial activity for employees aged 50 to 75 are the uncertainty of income, feeling old, and missing job security. These changes in opportunity perception at higher ages are, in turn, likely to negatively impact entrepreneurial activity. In other words, older people should favor salaried employment yielding instant payoffs over long-term returns from business ideas that would yet need to be implemented.

(1) *Hypothesis 4*: The negative relationship between age and entrepreneurial activity is mediated by perceived opportunities for entrepreneurship, such that age relates negatively to perceived opportunities, which in turn relate positively to entrepreneurial activity.

### The Role of Perceived Skills

The lifespan perspective (Baltes, 1987) proposes that various individual capabilities both increase and decrease at different rates over the course of time. One of the attributes that increases with age is crystallized intelligence (Salthouse, 2012). In regard to the work context, this increase in skills, knowledge, and experience might be attributed to higher job tenure and increased human, social, and financial capital. Thus, higher ages should relate to higher perceptions of entrepreneurial skills. The existence of agreement between broad actual skills and perceived skills was shown by Ackerman et al. (2002). They found that individuals generally tend to know their strengths and weaknesses in regard to different domains, including business and management.

Skills for entrepreneurship, however, do not solely comprise individuals’ crystallized cognitive abilities, as starting a venture requires the ability to recognize and exploit new opportunities, persistence to overcome potential obstacles, as well as physical strength and endurance to deal with likely stress in the starting phase and beyond. Based on the lifespan perspective, these other personal resources such as processing speed, memory, and physical endurance tend to decrease with age (Schulz and Heckhausen, 1996). As a consequence, people are less likely to be equipped with the necessary means to start new, future-oriented plans involving uncertainty as they age, leading to lower indications of *perceived skills* for entrepreneurship.

Moreover, with age people usually accumulate occupational, job, and organizational tenure, and therefore also higher task-related human capital, which could compensate for decreased physical abilities. However, when it comes to skills for entrepreneurship, physical abilities seem crucial. Thus, older adults might perceive that even though they have sufficient task-related human capital to work independently in their respective field of work, they may be relatively unequipped in regard to entrepreneurship (e.g., managing a business, handling stress, dealing with formalities). With increasing age, individuals might therefore perceive that they lack the skills relevant for entrepreneurship.

Conversely, younger adults might be biased regarding their own perceived skills and capabilities for entrepreneurship. As younger adults are more likely to be dissatisfied with the status quo (Charles, 2010), they are also more likely to suffer from self-enhancement bias (i.e., overconfidence), which builds on a personal need to increase personal satisfaction and self-worth (Lee and Im, 2007). We therefore assume that younger individuals are more likely to perceive themselves as having the necessary skills for entrepreneurship.

(1) *Hypothesis 5*: Age relates negatively to perceived skills for entrepreneurship.

As mentioned before, one of the recognitions that change across the lifespan may be the perception of own skills for entrepreneurship. The perception of own skills itself is strongly related to *self-efficacy*, which describes an individual’s belief to be capable of performing a given task (Gist, 1987). As self-efficacy builds on an individual’s assessment of own resources (Ajzen, 1987; Gist and Mitchell, 1992), it also relates to beliefs about goal-attainment. These beliefs, in turn, play a crucial role in the development of intentions and actions (Boyd and Vozikis, 1994). More specifically, only if individuals believe in having the necessary skills to attain a goal will they act upon it (Bandura, 1991).

Previous research has demonstrated the importance of self-efficacy, and thus the perception of skills, for entrepreneurial intention (e.g., Boyd and Vozikis, 1994; Chen et al., 1998; Zhao et al., 2005; Wilson et al., 2007). In a study by Chen et al. (1998), for example, an individual’s confidence in the ability to master entrepreneurial roles and tasks related positively to start-up intentions. Similarly, Zhao et al. (2005) found that the desire to become an entrepreneur is grounded in high entrepreneurial self-efficacy. As perceived skills for entrepreneurship resemble entrepreneurial self-efficacy (Bandura, 1993), we build on the aforementioned theorizing by proposing that perceived skills increase entrepreneurial activity itself, rather than merely intentions.

(1) *Hypothesis 6*: Perceived skills for entrepreneurship relate positively to entrepreneurial activity.

Taking the rationales for Hypotheses 5 and 6 together, we propose that perceived skills also mediate the relationship between age and entrepreneurial activity. First, age should be negatively associated with perceived skills due to certain age-related cognitive and physical declines. Second, this perception of having the necessary skills for entrepreneurship, or self-efficacy, should relate positively to entrepreneurial activity, as these perceptions are the foundation for goal pursuit.

(1) *Hypothesis 7*: The relationship between age and entrepreneurial activity is mediated by perceived skills for entrepreneurship, such that age relates negatively to perceived opportunities, which in turn relate positively to entrepreneurial activity.

## Method

### Participants and Procedure

Data for this study were based on the Global Entrepreneurship Monitor (GEM) from 2013, as it is the most recent publicly available dataset that is most likely to reflect current developments in entrepreneurship. The GEM is a global, standardized survey study. In 2013, it was administered to a representative sample of adults aged 16–98 (*M* = 40.55, *SD* = 14.19) in each participating country^1^ (*N* = 70), yielding a cross-country total of 244,471 participants. In each country, data collection was completed by professional firms, which were supervised by an academic or research institution. The project is coordinated by the Global Entrepreneurship Research Association (GERA), which monitors the data collection and secures standardization and international comparability of the data collection. The GEM data is frequently used in academic research, as it provides a “major database for internationally comparative entrepreneurship” (Bergmann et al., 2014, p. 1). The benefits of GEM data are its universality and comparability, which are based on the large number of national level observations which are comparable across countries (Ho and Wong, 2007; Langowitz and Minniti, 2007).

All procedures performed in this study were in accordance with the ethical standards of the 1964 Helsinki Declaration and its later amendments or comparable ethical standards. Because this study used existing data, which was collected by “National Teams” led by academic or research institutions that collected and reported the data, the GEM project was subject to their respective, ethical standards. Moreover, the data collection included a confidentiality note, so that no university ethical approval was required.

For this research, we excluded 3,661 participants (1.5%) due to missing values in some of the core variables, leading to a final sample size of 240,810 participants. Thereby, the sample sizes within the countries ranged from 33,287 in Turkey to 2,000 in Greece, Hungary, Norway, Poland, Malaysia, Singapore, Japan, South Korea, Lithuania, Latvia, Croatia, Macedonia, and Puerto Rico.

### Measures

Except for chronological age, all of the measures yielded binary answers (gender: 0 = *female*, 1 = *male*; all remaining variables: 0 = *no*, 1 = *yes*). Moreover, for reasons of practicability, most measures consisted of a single item. Yet, we argue that they are still valid measures, as the constructs they measure are rather clear and homogeneous. For example, Fisher et al. (2016) concluded that many single item measures can be used to assess several psychological constructs (e.g., work centrality, job control, life satisfaction) in a reliable and valid way.

#### Chronological Age

To assess age, survey respondents were asked to indicate their exact age in years at the time of the interview.

#### Entrepreneurial Activity

We used entrepreneurial activity instead of entrepreneurial intentions as our outcome variable, because we see entrepreneurial activity as the desired outcome of business creation. While intentions can be seen as an antecedent of entrepreneurial activity, it was not possible to use intentions and activity in the same model, as (a) individuals participating in GEM cannot be matched across measurement waves and (b) it is not possible to predict entrepreneurial activity from intentions using measures collected at the same point in time.

Entrepreneurial activity was measured by total early-stage entrepreneurial activity (TEA), consisting of nascent entrepreneurs involved in setting up a business, as well as owners of a new firm being less than 42 months old. It was assessed by asking “Are you, alone or with others, currently trying to start a new business, including any self-employment or selling any goods or services to others?” and “Are you, alone or with others, currently trying to start a new business or a new venture for your employer as part of your normal work?” Participants replying “yes” to either item were categorized as engaging in entrepreneurial activity (i.e., 1), while participants replying “no” to both items were categorized as not engaging in entrepreneurial activity (i.e., 0). Moreover, it was required that participants had conducted concrete actions over the past 12 months, and were one of the owners, or the sole owner, of the business-in-gestation. If businesses were older than 42 months, participants were characterized as a manager of either a new or an established firm, depending on whether financial payments had been made for longer than 42 months (i.e., established firm) or less (i.e., new firm).

The TEA index has been frequently used in academic research (see Reynolds et al., 2004; Ho and Wong, 2007; Levie and Autio, 2008; McMullen et al., 2008, for examples). For instance, in their study on financing, regulatory costs and entrepreneurial propensity, Ho and Wong (2007) found that overall TEA related significantly and positively to informal investors as a funding source for start-ups (*r* = 0.52).

#### Perceived Opportunities

To assess perceived opportunities, respondents were asked: “In the next 3 months, will there be good opportunities for starting a business in the area where you live?” Since this operationalization focuses on business opportunity identification (Shane and Venkataraman, 2000), it is slightly different from the concept “focus on opportunities” from the lifespan literature (Zacher et al., 2010). While focus on opportunities describes the number of remaining opportunities for oneself, perceived opportunities relate to general business opportunity identification. Yet, due to the link between declining fluid intelligence (e.g., processing speed) in old age and opportunity identification (Krueger and Welpe, 2014; Helfat and Peteraf, 2015), older individuals are likely to be less able to identify opportunities compared to younger individuals. Thus, they are also likely to see less opportunities remaining.

This scale was previously used in academic research (e.g., Langowitz and Minniti, 2007; Levie and Autio, 2008). For example, Langowitz and Minniti (2007) found that perceived opportunities were significantly and positively related to the likelihood of being a nascent entrepreneur (*r* = 0.14).

#### Perceived Skills

For perceived skills, participants were asked to indicate if they “… have the knowledge, skill and experience required to start a new business?” This scale was previously validated in academic research (e.g., Langowitz and Minniti, 2007; Levie and Autio, 2008). In the aforementioned study by Langowitz and Minniti (2007), perceived skills were significantly and positively related to the likelihood of being a nascent entrepreneur (*r* = 0.20).

#### Control Variables

We controlled for gender (0 = *female*, 1 = *male*), as well as the country participants resided in by means of a multilevel analysis. Wilson et al. (2007) showed that women are more likely to limit their career choices due to lacking confidence in their abilities, which is especially influential in regard to entrepreneurial activity (Chen et al., 1998).

### Statistical Analysis

We used logistic path analysis in Mplus (Muthén and Muthén, 2012) to test our hypotheses. Due to the large amount of data coming from different countries, we first checked for random slopes to determine the type of analysis using Mplus. As there was not sufficient variation between countries, we continued with the analysis using fixed slopes. Specifically, we proceeded with the main analysis on the within-level (i.e., person-level) only. Even though we hypothesized linear relations, we included age-squared^2^ in our analysis to check for curvilinearity to account for potential non-linear relationships, as recommended by Bohlmann et al. (2017).

As the dependent variables were binary, the WLSMV estimator was used to estimate both main and indirect effects. Its robustness with non-normally distributed variables has been shown previously, and makes it the best option for modeling ordered or categorical data (Brown, 2006). This model builds on the principles of general linear models, but allows for a better account of dichotomous dependent variables. Moreover, probit models extend the standard log-linear model, thereby allowing for mixtures of both categorical and continuous independent variables and their relation to a categorical outcome. Another advantage of probit models is that the resulting regression coefficients can be interpreted as the change in probabilities when the binary variable changes from 0 to 1 (Langowitz and Minniti, 2007).

Differences between countries were accounted for by using “TYPE = COMPLEX” to ensure the validity and reliability of the results. In the first model, we examined the direct effect of age and age-squared on entrepreneurial activity while including gender as a control variable. In the second model, we added the two mediator variables to examine mediation effects (i.e., ran a single model with multiple mediators). To further investigate the mediation effects, we checked whether the significant, direct effects of age on entrepreneurial activity became insignificant after including the mediators to the model (i.e., full mediation), or whether direct, indirect, and total effects were all significant (i.e., partial mediation).

## Results

**FIGURE 2:**
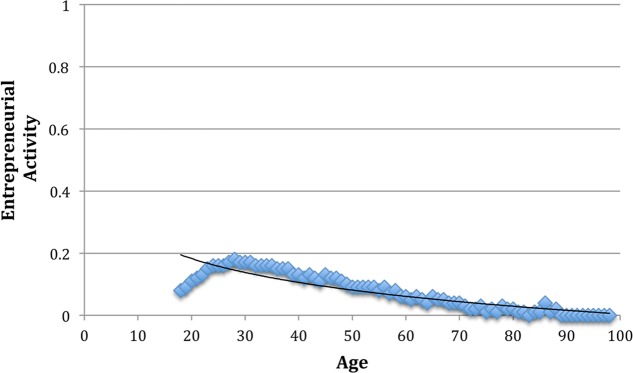
Plot of entrepreneurial activity across the lifespan (18–97 years). The trendline depicts the logarithmic relationship between age and entrepreneurial activity.

Descriptive statistics and correlations of the variables are shown in **Table 1**. Of note, all correlations were significant (*p* < 0.01) due to the large sample size. Yet, correlations among predictors were relatively small, hinting at low overlap between variables. The remaining results are displayed in **Tables 2** and **3**. In Model 1 (pseudo *R*^2^ = 0.07, *p* < 0.001), age (β = -0.20, *p* < 0.001) and age-squared (β = -0.15, *p* < 0.001) significantly predicted entrepreneurial activity (**Table 2**). The results are in line with Hypothesis 1, which states that the relationship between age and entrepreneurial activity is negative. To further investigate the curvilinear pattern, we plotted the results (**Figure 2**), showing that the negative relation weakened somewhat at higher ages.

**Table 1:** Descriptive statistics and correlations.

Variables	*M*	*SD*	1	2	3	4	5	6
(1) Age	40.55	14.19	–	0.00	-0.08^∗∗^	-0.05^∗∗^	-0.09^∗∗^	-0.02^∗∗^
(2) Age-squared	0.00	232.82		–	0.02^∗∗^	-0.10^∗∗^	-0.05^∗∗^	-0.02^∗∗^
(3) Perceived opportunities for entrepreneurship^a^	0.40	0.49			–	0.22^∗∗^	0.18^∗∗^	0.05^∗∗^
(4) Perceived skills for entrepreneurship^a^	0.52	0.50				–	0.24^∗∗^	0.13^∗∗^
(5) Entrepreneurial activity^a^	0.12	0.32					–	0.05^∗∗^
(6) Gender^b^	0.51	0.50						–


**N* ranges from 208,475 to 240,817. ^a^Response format was 0 = no, 1 = yes. ^b^Response format was 0 = female, 1 = male. ^∗∗^*p* < 0.01.*

**Table 2:** Results of regression analyses.

	Entrepreneurial activity					
					
	Model 1		Model 2		Perceived opportunities		Perceived skills	
				
Predictor variables	Coefficient	*SE*	Coefficient	*SE*	Coefficient	*SE*	Coefficient	*SE*
Age	-0.199^∗∗∗^	0.014	-0.137^∗∗∗^	0.011	-0.102^∗∗∗^	0.017	-0.057^∗∗^	0.018
Age squared	-0.148^∗∗∗^	0.011	-0.092^∗∗∗^	0.011	0.055^∗∗^	0.020	-0.148^∗∗∗^	0.013
Perceived opportunities for entrepreneurship			0.333^∗∗∗^	0.025				
Perceived skills for entrepreneurship			0.487^∗∗∗^	0.017				
Gender	0.082^∗∗∗^	0.012	-0.014	0.009	0.059^∗∗∗^	0.008	-0.157^∗∗∗^	0.010
Pseudo *R^2^*	0.069^∗∗∗^		0.403^∗∗∗^		0.017^∗∗∗^		0.051^∗∗∗^	


**N* = 240,810. All values are standardized. ^∗∗^*p* < 0.01, ^∗∗∗^*p* < 0.001.*

**Table 3:** Standardized indirect effects of age and age squared on entrepreneurial activity.

Hypotheses	Coefficient	Standard error
Age → Perceived Opportunities for Entrepreneurship → Entrepreneurial Activity	-0.034^∗∗^	0.007
Age → Perceived Skills for Entrepreneurship → Entrepreneurial Activity	-0.028^∗∗^	0.009
Age Squared → Perceived Opportunities for Entrepreneurship → Entrepreneurial Activity	0.018^∗^	0.007
Age Squared → Perceived Skills for Entrepreneurship → Entrepreneurial Activity	-0.072^∗∗^	0.006


**N* = 240,810. The displayed estimates are based on a single mediation model. Perceived opportunities, perceived skills, and entrepreneurial activity were coded 0 = no, 1 = yes. ^∗^*p* < 0.05, ^∗∗^*p* < 0.01.*

**FIGURE 3:**
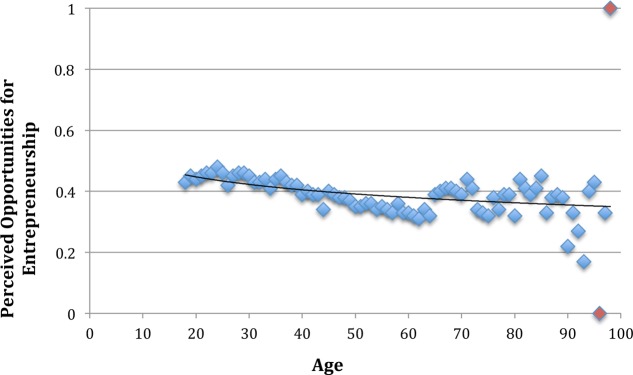
Plot of perceived opportunities for entrepreneurship across the lifespan (16–97 years). Red dots indicate extreme age groups, consiting of very few observations (i.e., *N* = 2 for age 96, *N* = 1 for age 98). The trendline depicts the logarithmic relationship between age and perceived opportunities for entrepreneurship.

Adding perceived opportunity and skills significantly improved the model, yielding to a total pseudo *R*^2^ of 0.40 (*p* < 0.001; Model 2 in **Table 2**). Hypothesis 2 states that the relation between age and the perception of opportunities is negative. As seen in **Table 2**, age was significantly and negatively related to perceived opportunities (β = -0.10, *p* < 0.001), thereby confirming Hypothesis 2. As seen in **Figure 3**, this relation was also weakly curvilinear in nature, as age-squared also significantly predicted perceived opportunities (β = 0.06, *p* = 0.005).

Hypothesis 3 states that perceived opportunities are positively related to entreneurial activity. Based on the results in **Table 2**, perceived opportunities did indeed relate postively and significantly to entrepreneurial activity (β = 0.33, *p* < 0.001). This confirms Hypothesis 3.

Hypothesis 4 stated that the negative relationship between age and entrepreneurial activity is mediated by perceived opportunities. As shown in **Table 3**, results supported this indirect effect (-0.03, *p* < 0.001). However, as the effect of age remained significant (β = -0.14, *p* < 0.001), the mediation is only partial.

**FIGURE 4:**
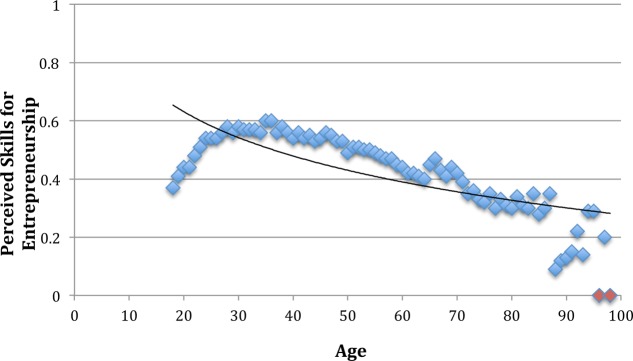
Plot of perceived skills for entrepreneurship across the lifespan (16–97 years). Red dots indicate extreme age groups, consiting of very few observations (i.e., *N* = 2 for age 96, *N* = 1 for age 98). The trendline depicts the logarithmic relationship between age and perceived opportunities for entrepreneurship.

Hypothesis 5 states that the relation between age and the perception of skills is negative. As seen in **Table 2**, this hypothesis was confirmed as age related negatively and significantly to the perception of skills (β = -0.06, *p* = 0.002). Additionally, as seen in **Figure 4**, this relationship was weakly curvilinear (β = -0.15, *p* < 0.001).

Hypothesis 6 states that the relation between perceived skills and entrepreneurial activity is positive. Results in **Table 2** confirm this, as perceived skills related positively and significantly to entrepreneurial activity (β = 0.49, *p* < 0.001).

Hypothesis 7 states that the negative relationship between age and entrepreneurial activity is mediated by perceived skills. **Table 3** shows that the indirect effect was significant (-0.03, *p* = 0.001), lending support for Hypothesis 7. As the effect of age remained significant (β = -0.14, *p* < 0.001), the mediation is only partial.

## Discussion

### Summary and Interpretation of Findings

In this article, we adopted a lifespan perspective to uncover how age relates to entrepreneurial activity through individuals’ perceptions of entrepreneurial opportunities and skills. In accordance with the hypotheses, results showed that there is a negative relation between age and entrepreneurial activity. Moreover, this relation was partially mediated by perceived opportunities and perceived skills for entrepreneurship. Specifically, age related negatively to both perceived opportunities and skills. Perceived opportunities and skills, in turn, related positively to entrepreneurial activity.

The relations between age and entrepreneurial activity, as well as between age and both perceived opportunities and perceived skills, were weakly curvilinear (i.e., inversely U-shaped). While this was not part of our hypotheses, the decline in entrepreneurial activity with age might be less steep in middle adulthood, as these individuals are less likely to be aware of their future time perspective due to higher commitments in regard to both career and family, as compared to younger or older adults. Moreover, the steeper decline in perceived skills in later years might be due to the fact that the decline in both cognitive (Verhaeghen and Salthouse, 1997) and physical abilities (Spirduso et al., 1995) accelerates in late adulthood.

While the results are of statistical significance, they are practically relevant as well. More specifically, the pseudo R-square indicated that 40% of the variance in entrepreneurial activity can be explained by the probit analysis (McKelvey and Zavoina, 1975). Therefore, the results are a first step toward a more comprehensive conceptualization of the entrepreneurial process from a lifespan perspective. They are especially important as previous research in entrepreneurship has assumed an influence of age, but mostly controlled for it, instead of adding it as a substantive predictor of entrepreneurial activity (Schjoedt and Bird, 2014). Consequently, the findings have important implications for entrepreneurship theories. First, major theories have conceptualized entrepreneurial activity as timeless. For example, according to the theory of planned behavior (Ajzen and Fishbein, 1977), attitudes toward a given behavior, subjective norms about the appropriateness and desirability of the behavior, as well as perceived control about the behavior determine the exertion of a given behavior, such as entrepreneurial activity. Yet, the current study confirmed the importance of age by showing that entrepreneurial activity changes over time and at different rates. Second, by including perceived opportunities and skills as mediators, the present study hints at the ways through which age exerts its influence. Current theories used in entrepreneurship (e.g., the theory of planned behavior; Ajzen and Fishbein, 1977) can therefore not accurately predict entrepreneurial activity, as they do not account for changes in individual skills and opportunities that are due to age and age-related characteristics. It is thus crucial to amend current models of entrepreneurship by adding age as a predictor. However, it has to be noted that age itself cannot be directly related to entrepreneurial activity, as it is mostly a proxy variables that helps to measure time-related changes as people age (Wohlwill, 1970). Thus, it is important to investigate mediators, such as perceived opportunities and perceived skills for entrepreneurship.

Next to the theoretical implications, the results have practical implications as well. Findings suggest that younger and older adults need to be supported differently throughout the entrepreneurial process. While younger adults have less difficulty to perceive opportunities and acknowledge that they are skilled enough to act entrepreneurially, older adults may be constrained by a lower future time-perspective and declining physical and fluid cognitive abilities, and thus less likely to perceive opportunities and skills. It might therefore be important to help younger adults acquire the relevant skills to act on their identified opportunity, while older adults might need help finding opportunities that require less time to reach the desired goal. This knowledge is especially important for governments and educational institutions, as these usually employ training programs to foster entrepreneurship (Obschonka et al., 2010; Halvorson and Morrow-Howell, 2016). These programs currently do not accurately take the needs and motivations of different ages into account.

### Limitations and Future Research

A central limitation of this study is the cross-sectional nature of the data, which does not allow for a separation of cohort effects and age-related change (Schaie, 1965). Moreover, there may be reverse effects, as the definite direction of the hypothesized relations can only be confirmed in a longitudinal study. It might thus be that entrepreneurial activity increases perceived opportunities (i.e., because people are in the entrepreneurial mindset once they engaged in entrepreneurship and therefore constantly perceive new opportunities) and perceived skills (i.e., because performance can enhance self-efficacy perceptions; Sitzmann and Yeo, 2013). However, cross-sectional studies still provide valuable information on systematic age differences (Lévesque and Minniti, 2006; Kautonen et al., 2010; Gielnik et al., 2012; Caliendo et al., 2014; Heim, 2015) that can be amended and replicated in other studies employing different designs.

Another limitation is the binary nature of most study variables, which might restrict a continuous assessment of the respective variables. However, the aim of the global research project (GEM) is to collect representative data in as many countries as possible, which requires keeping the questionnaire relatively short and avoiding answer options that might lead to translation errors or cultural biases. Moreover, the validity of measures used has been established in previous studies (Bergmann et al., 2014). Thus, results can still yield preliminary insights on what influences the changing relation between age and entrepreneurial activity.

To validate results of the few studies that have yet investigated relationships between age and entrepreneurship, future research should employ different designs that can help to determine both developments over time as well as causality (e.g., by using longitudinal or experimental designs; Bohlmann et al., 2017). While opportunities and skills are some of the concepts through which age and age-related characteristics impact the entrepreneurial process, future studies need to research other individual and contextual factors that play a role for entrepreneurship while being influenced by age. For example, investigating whether age-differences are the same or different across cultures can help to isolate developmental change across the lifespan from culture-related cohort effects (McCrae et al., 1999). Other examples are push factors of entrepreneurship, such as unemployment in older age (e.g., Faria et al., 2010).

Moreover, the time remaining relative to one’s age is not only shaped by individual, chronological age, but also tied to the average life expectancy of a given country (Seijts, 1998; Griffin et al., 2016). It is therefore important to consider variations in life expectancies across countries as an influence of an individual’s future-time perspective when investigating the impact of age on perceived opportunities and, in turn, entrepreneurship. More specifically, if the life expectancy in a given country is relatively short, the future time perspective of a respondent from this country is likely to be lower compared to a respondent from a country with the same age and a higher average life expectancy. In regard to an individual’s context, cultural variables that merge characteristics of regional infrastructure as well as the social and economic environment are likely to shape entrepreneurial activity as well. Thereby, factors such as institutional support or aging stereotypes seem to be especially impactful (e.g., Delmar and Davidson, 2000; Thomas and Muller, 2000; Kennedy et al., 2003).

## Conclusion

The present study takes a lifespan perspective on entrepreneurial activity. Although the data does not allow for the identification of casual relationships, results suggest that perceptions of opportunities and skills for entrepreneurship are related to entrepreneurial activity, and can help to better understand the role of age and age-related changes for entrepreneurship. Ultimately, economic models of entrepreneurial activity should include age and potential mediators of its relationship with entrepreneurial activity. In practice, institutions can use the results to gain the necessary knowledge to successfully foster entrepreneurship as a means of financial security and employment for people from different age groups.

## Author Contributions

CB: conception and design, data analysis and interpretation, drafting the article, critical revision of the article. AR: conception and design, helped with data analysis and interpretation, critical revision of the article. HZ: conception and design, critical revision of the article.

## Conflict of Interest Statement

The authors declare that the research was conducted in the absence of any commercial or financial relationships that could be construed as a potential conflict of interest.

## Footnotes

1 Included countries were: United States, Russia, South Africa, Greece, Netherlands, Belgium, France, Singapore, Thailand, Japan, South Korea, Vietnam, China, Turkey, India, Iran, Canada, Spain, Hungary, Italy, Romania, Switzerland, United Kingdom, Sweden, Norway, Poland, Germany, Peru, Mexico, Argentina, Brazil, Chile, Colombia, Malaysia, Indonesia, Philippines, Algeria, Libya, Ghana, Nigeria, Angola, Barbados, Uganda, Zambia, Malawi, Botswana, Namibia, Portugal, Luxembourg, Ireland, Finland, Lithuania, Latvia, Estonia, Croatia, Slovenia, Bosnia and Herzegovina, Macedonia, Czechia, Slovakia, Guatemala, Panama, Ecuador, Suriname, Uruguay, Puerto Rico, Trinidad and Tobago, Jamaica, and Taiwan.

2 To obtain a measure of age-squared that is uncorrelated with age, we first ran a regression with age as the predictor variable and age-squared as the outcome variable, and saved the unstandardized residuals as a new variable. The unstandardized residuals were used as age-squared in the analysis.
